# Inflammatory-nutritional duality of NPAR: a novel biomarker for early prediction of acute liver injury in acute myocardial infarction complicated by type 2 diabetes mellitus

**DOI:** 10.3389/fphar.2025.1643871

**Published:** 2025-09-02

**Authors:** Xiaorui Huang, Zizhu Lian, Shanshan Qi, Hang Yu

**Affiliations:** ^1^ Department of Cardiology, The First Affiliated Hospital of Xi’an Jiaotong University, Xi’an, China; ^2^ Department of Cardiovascular Surgery, The First Affiliated Hospital of Xi’an Jiaotong University, Xi’an, China; ^3^ Department of Health Sciences, The First Affiliated Hospital of Xi’an Jiaotong University, Xi’an, China

**Keywords:** acute myocardial infarction (AMI), acute liver injury (ALI), type 2 diabetes mellitus (T2DM), neutrophil percentage-to-albumin ratio (NPAR), cancer

## Abstract

**Background/Objectives:**

Acute liver injury (ALI) is a severe complication of acute myocardial infarction (AMI) comorbid with type 2 diabetes mellitus (T2DM), serving as an independent risk factor for adverse prognosis and imposing a significant disease burden. The aim of this study was to identify predictive value of a composite of inflammation and nutrition-related indicators for the risk of ALI in AMI patients comorbid with T2DM.

**Methods:**

AMI patients with T2DM hospitalized at the First Affiliated Hospital of Xi’an Jiaotong University between January 2018 and May 2025 were included. Clinical data and medication information were collected through the hospital’s biospecimen information resource center. The patients enrolled were divided into non-hepatic injury group, mild hepatic injury group and moderate-to-severe hepatic injury group according to the alanine transaminase (ALT) level tested during hospitalization. Neutrophil percentage-to-albumin ratio (NPAR) was calculated as the ratio of neutrophil percentage (NP) to serum albumin (ALB) level (NPAR = NP/ALB). The primary outcome is acute liver injury during hospitalization.

**Results:**

Among 5133 AMI patients with T2DM (76.57% male, median age 62.61 years (51.29–72.93), acute liver injury occurred in 7.60% (n = 390) of the cohort and moderate-to-severe hepatic injury occurred in 1.34% (n = 69). Compared with non - hepatic injury, mild (60.7% vs. 45.2%, *P* < 0.001) and moderate-to-severe hepatic injury (65.2% vs. 45.2%, *P* < 0.001) had higher ST - segment elevation myocardial infarction (STEMI) rates. Both hepatic injury subgroups showed higher Killip III/IV prevalence (mild: 26.2% vs. 8.7%, moderate-to-severe: 53.6% vs. 8.7%, both *P* < 0.001). Moderate-to-severe vs. mild injury had older age (69.50 ± 11.79 vs. 64.35 ± 11.77, *P* < 0.05) and more arrhythmias (46.4% vs. 27.1%, *P* < 0.001). After adjustment, NPAR independently predicted ALI (OR = 1.70, 95% CI: 1.33–2.17) and moderate-to-severe injury (OR = 2.90, 95% CI: 1.90–4.42), with an AUC of 0.86 (95% CI: 0.81–0.92) for moderate-to-severe injury. A history of cancer was an independent risk factor for ALI. Among these patients, NPAR demonstrated AUCs of 0.83 for overall ALI and 0.89 for moderate-to-severe ALI.

**Conclusion:**

NPAR effectively predicts moderate-to-severe hepatic injury in AMI patients with T2DM, suggesting its potential as a clinically useful early marker. Furthermore, while cancer is an independent risk factor for ALI in this population, NPAR maintains strong predictive performance for ALI even in this high-risk subgroup.

## 1 Introduction

Acute myocardial infarction (AMI) complicated by type 2 diabetes mellitus (T2DM) constitutes a high-risk clinical entity characterized by elevated morbidity and mortality, imposing substantial clinical and socioeconomic burdens (Mi et al., 2023; Karayiannides et al., 2018). While advancements in diagnosing and therapeutic interventions (Collet et al., 2021), multiorgan dysfunction - particularly acute liver injury (ALI) - represents a critical mediator of adverse outcomes in this population (Ford et al., 2017; Gao et al., 2017) and worsens the clinical outcomes.

The hepatic vulnerability in AMI-T2DM patients arises from dual pathophysiological insults: hemodynamic compromise due to impaired cardiac output and diabetes-associated metabolic derangements (chronic hyperglycemia, oxidative stress, and systemic inflammation) (Vollmar and Menger, 2009). This synergistic injury cascade precipitates hepatocellular dysfunction, exacerbating systemic metabolic decompensation through impaired detoxification and gluconeogenic regulation, ultimately amplifying clinical deterioration (Gao et al., 2017).

Emerging evidence highlights neutrophil percentage-to-albumin ratio (NPAR) as a novel prognostic biomarker integrating inflammatory activity and nutritional-metabolic status (Fu and Feng, 2024; Lin et al., 2022). Given neutrophils’ role in oxidative tissue damage and hypoalbuminemia’s association with systemic metabolic dysregulation - both central to ALI pathogenesis. We hypothesize NPAR may enable early risk stratification of ALI in AMI-T2DM patients. This study investigates predictive value of NPAR in this vulnerable cohort, aiming to optimize hepatoprotective strategies through cost-effective biomarker guidance.

## 2 Results

### 2.1 Characteristics of the study cohort

Ultimately, this study enrolled 5,133 subjects, among whom 101 (1.97%) were cancer patients, the median age was 62.61 years (IQR: 51.29, 73.93), including 3,915 (76.27%) male patients. 390 (7.60%) of them occurred ALI and 69 (1.34%) of them occurred moderate-to-severe hepatic injury (Table 1). Compared to the non-hepatic injury group, the hepatic injury groups exhibited significantly higher proportion of ST-segment elevation myocardial infarction (STEMI) with 195 cases (60.7%) in the mild group and 45 cases (65.2%) in the moderate-to-severe group vs. 2,134 cases (45.2%) in the non-injury group (*P* < 0.001), and the moderate-to-severe hepatic injury group showed a greater prevalence of Killip III/IV with 37 cases (53.6%) versus 413 cases (8.7%) in the non-injury group (*P* < 0.001). Compared to the mild hepatic injury subgroup, the moderate-to-severe hepatic injury group demonstrated older age (69.50 ± 11.79 years vs. 64.35 ± 11.77 years, *P* < 0.05), a higher rate of arrhythmias (32 cases [46.4%] vs. 87 cases [27.1%], *P* < 0.001), and a decreased proportion of anterior wall infarction (4 cases [5.8%] vs. 51 cases [15.9%], *P* < 0.05). Additionally, the hepatic injury groups showed increased utilization of diuretics with 152 cases (47.4%) in the mild group and 25 cases (36.2%) in the moderate-severe group versus 1,502 cases (31.8%) in the non-injury group (*P* < 0.001), but reduced use of lipid-lowering agents with 264 cases (82.2%) in the mild group and 31 cases (45.0%) in the moderate-to-severe group vs. 4,544 cases (96.2%) in the non-injury group (*P* < 0.001) (Table 2). Patients with moderate-to-severe hepatic injury paradoxically had a lower prevalence of hypertension history (49.3% vs. 61.7% and 64.6%, *P* = 0.020).

**TABLE 1:** Demographic characteristics of AMI patients stratified by acute liver injury.

Variables	Non-hepatic injury group (ALT < 2ULN) n = 4,724	Mild hepatic injury group (2ULN ≤ ALT < 5ULN) n = 321	Moderate-to-severe hepatic injury group (ALT ≥ 5ULN) n = 69	P
age,year	62.69 ± 11.28	64.35 ± 11.77	69.50 ± 11.79^a^	0.017
male, n (%)	3,614 (76.5%)	250 (77.9%)	51 (73.9%)	0.749
Hx of HTN, n (%)	3,049 (64.6%)	198 (61.7%)	34 (49.3%)^b^	0.020
Hx of stroke, n (%)	602 (12.7%)	32 (10.0%)	5 (7.2%)	0.143
Hx of AMI, n (%)	406 (8.6%)	24 (7.5%)	7 (10.1%)^b^ ^,^ ^a^	0.701
Hx of kidney disease, n (%)	395 (8.4%)	22 (6.9%)	9 (13.0%)	0.231
Arrhythmia, n (%)	730 (15.5%)	87 (27.1%)^b^	32 (46.4%)^b^ ^,^ ^a^	<0.001
Cancer, n (%)	78 (1.7%)	16 (5.0%)	7 (10.1%)	<0.001
SBp, mmHg	124.51 ± 20.87	118.11 ± 20.09^b^	115.35 ± 21.42^b^	<0.001
DBp, mmHg	75.35 ± 13.12	73.94 ± 12.36	71.58 ± 15.03	0.014
HR, bpm	78.09 ± 14.19	80.91 ± 17.57	77.82 ± 20.43	0.004
STEMI, n (%)	2,134 (45.2%)	195 (60.7%)^b^	45 (65.2%)^b^ ^,^ ^a^	<0.001
Killip III/IV	413 (8.7%)	84 (26.2%)	37 (53.6%)^b^	<0.001
Hospital stay, day	4.38 (2.73, 6.44)	5.09 (3.03, 8.45)	6.46 (3.35, 10.27)	0.176

^a^ compared with mild hepatic injury group: *P* < 0.05.

^b^ compared with non-hepatic injury group: *P* < 0.05.

Abbreviation: Hx: History, HTN: hypertension, SBp: Systolic Blood pressure, DBp: Diastolic Blood pressure, HR: heart rate, bpm: beats per minute.

**TABLE 2:** Characteristics of coronary angiography and treatment of AMI patients.

Variables	Non-hepatic injury group (ALT < 2ULN) n = 4,724	Mild hepatic injury group (2ULN ≤ ALT < 5ULN) n = 321	Moderate-to-severe hepatic injury group (ALT ≥ 5ULN) n = 69	*P*
Infarction location				<0.001
Anterior	870 (18.4%)	51 (15.9%)^a^	4 (5.8%)^a^ ^,^ ^b^	
Extensive Anterior	397 (8.4%)	46 (14.3%)^a^	12 (17.4%)^a^ ^,^ ^b^	
Posterior	439 (9.3%)	54 (16.8%)	8 (11.6%)	
(high) Lateral	99 (2.1%)	15 (4.7%)	10 (14.5%)	
Inferior	694 (14.7%)	40 (12.5%)	12 (17.4%)	
RVMI	57 (1.2%)	8 (2.5%)	4 (5.8%)	
Diseased vessels				0.724
Single, n (%)	482 (10.2%)	29 (9.0%)	5 (7.2%)	
Dual, n (%)	1,020 (21.6%)	78 (24.3%)	17 (24.6%)	
Triple, n (%)	3,222 (68.2%)	214 (66.7%)	47 (68.1%)	
Treatment				0.312
Medicine, n (%)	132 (2.68%)	8 (2.5%)	4 (5.8%)	
PCI/PTCA, n (%)	4,544 (96.2%)	307 (95.6%)	63 (91.3%)	
CABG, n (%)	48 (1.0%)	6 (1.9%)	2 (2.9%)	
Medicine				
Antiplatelet drug				<0.001
Single, n (%)	99 (2.1%)	8 (2.5%)	8 (11.6%)	
Dual, n (%)	4,498 (95.2%)	277 (86.3%)	17 (50.7%)	
β-blocker, n (%)	2,948 (62.4%)	203 (63.2%)	49 (71.0%)	0.643
ACEI/ARB, n (%)	2,234 (47.3%)	123 (41.1%)	16 (23.2%)^a^	0.036
CCB, n (%)	505 (10.7%)	19 (5.9%)	8 (11.6%)	0.219
Nitrate, n (%)	2,562 (11.9%)	24 (7.5%)	8 (11.6)	0.144
Diuretic, n (%)	1,502 (31.8%)	152 (47.4%)^a^	25 (36.2%)^a^ ^,^ ^b^	<0.001
Statin, n (%)	4,544 (96.2%)	264 (82.2%)^a^	31 (45.0%)^a^ ^,^ ^b^	<0.001
ARNI, n (%)	1,068 (22.6%)	62 (19.3%)	23 (33.3%)^a^	0.023
s-GLT2i, n (%)	1767 (37.4%)	103 (32.1%)	12 (17.4%)	0.782

^a^ compared with non-hepatic injury group: *P* < 0.05.

^b^ compared with mild hepatic injury group: *P* < 0.05.

Abbreviation: RVMI: right ventricular myocardial infarction, PCI: percutaneous coronary intervention, PTCA: percutaneous transluminal coronary angioplasty, CABG: coronary artery bypass grafting, ACEI: Angiotensin-Converting Enzyme Inhibitor, ARB: Angiotensin II, receptor blocker, CCB: calcium channel blocker, ARNI: angiotensin receptor neprilysin inhibitor, sGLT2i: Sodium-Glucose Cotransporter-2, inhibitor.

As shown in Table 3, patients with hepatic injury demonstrated significantly elevated levels of creatine kinase-MB (CK-MB: mild 24.00 [13.00, 80.75] U/L vs. non-injury 19.00 [12.17, 44.31] U/L), creatine kinase (CK: mild 231.00 [82.00, 1,259.50] U/L vs. non-injury 138.00 [73.00, 450.00] U/L), cardiac troponin T (cTnT: mild 0.67 [0.12, 3.38] ng/mL vs. non-injury 0.24 [0.05, 0.94] ng/mL), N-terminal pro-B-type natriuretic peptide (NT-proBNP: mild 1,491.00 [293.50, 4,645.00] pg/mL vs. non-injury 814.35 [247.28, 2,560.50] pg/mL), high-sensitivity C-reactive protein (hs-CRP: mild 5.82 ± 3.62 mg/L vs. non-injury 4.89 ± 3.84 mg/L), random blood glucose on admission (Glu: mild 11.22 ± 5.40 mmol/L vs. non-injury 10.34 ± 4.55 mmol/L), and D-dimer (D-D: mild 0.86 [0.55, 2.07] mg/L vs. non-injury 0.62 [0.42, 1.00] mg/L), with these parameters markedly higher in the moderate-to-severe subgroup than in the mild hepatic injury subgroup. In contrast, low-density lipoprotein cholesterol (LDL-C: mild 2.26 ± 0.87 mmol/L vs. non-injury 2.32 ± 0.92 mmol/L), partial pressure of carbon dioxide (PCO2: mild 36.23 ± 6.63 mmHg vs. non-injury 36.97 ± 5.18 mmHg), estimated glomerular filtration rate (eGFR: mild 74.99 ± 20.60 mL/min/1.73 m^2^ vs. non-injury 76.60 ± 20.85 mL/min/1.73 m^2^), prothrombin activity (PTA: mild 88.89% ± 21.56% vs. non-injury 97.54% ± 18.56%), hemoglobin (mild 136.34 ± 21.91 g/L vs. non-injury 135.41 ± 21.56 g/L), and serum albumin (mild 37.58 ± 5.33 g/L vs. non-injury 38.52 ± 5.53 g/L) were significantly lower in the hepatic injury groups, with these differences further amplified in the moderate-to-severe subgroup compared to the mild hepatic injury subgroup.

**TABLE 3:** Comparison of clinical characteristics and laboratory parameters of AMI patients.

Variables	Non-hepatic injury group (ALT < 2ULN) n = 4,724	Mild hepatic injury group (2ULN ≤ ALT < 5ULN) n = 321	Moderate-to-severe hepatic injury group (ALT ≥ 5ULN) n = 69	*P*
CKMB, U/L	19.00 (12.17, 44.31)	24.00 (13.00, 80.75)^a^	32.26 (19.90, 109.75)^a^	<0.001
CK, U/L	138.00 (73.00, 450.00)	231.00 (82.00, 1,259.50)^a^	569.00 (112.25, 2,707.75)^a^	<0.001
cTnT, ng/mL	0.24 (0.05, 0.94)	0.67 (0.12, 3.38)^a^	2.16 (0.48, 4.43)^a^	<0.001
NT-proBNP, pg/mL	814.35 (247.28, 2,560.50)	1,491.00 (293.50, 4,645.00)^a^	3,167.50 (932.50, 10,653.25)^a,b^	<0.001
hs-CRP, mg/L	4.89 ± 3.84	5.82 ± 3.62^a^	7.32 ± 3.83	<0.001
CHOI	4.07 ± 1.22	3.96 ± 1.14	3.63 ± 1.19	0.003
LDL-C, mmol/L	2.32 ± 0.92	2.26 ± 0.87	2.00 ± 0.90^a,b^	0.016
HDL-C, mmol/L	0.92 ± 0.23	0.89 ± 0.22	0.92 ± 0.26^a^	0.298
Apo A	1.04 ± 0.19	0.99 ± 0.21	0.90 ± 0.24	<0.001
Apo B	0.80 ± 0.24	0.77 ± 0.26	0.68 ± 0.25	0.001
HbA1c, %	8.03 ± 1.74	7.86 ± 1.74^a^	8.45 ± 2.30	0.043
Glu, mmol/L	10.34 ± 4.55	11.22 ± 5.40	13.68 ± 6.82	<0.001
Na+, mmol/L	137.19 ± 3.88	136.98 ± 4.67	133.69 ± 8.09^a,b^	0.059
K+, mmol/L	3.72 ± 0.42	3.82 ± 0.48	3.88 ± 0.61^a^	0.095
Mg2+, mmol/L	0.97 ± 0.13	1.01 ± 0.15^a^	1.16 ± 0.48^a,b^	<0.001
P-, mmol/L	1.00 ± 0.29	1.02 ± 0.32	1.47 ± 0.59^a,b^	<0.001
BUN, mmol/L	7.06 ± 4.03	7.99 ± 4.30	13.03 ± 9.26	<0.001
CRE, mmol/L	64.00 (53.00, 80.00)	71.00 (55.25, 91.00)^a^	94.00 (76.00, 117.00)^a^	<0.001
eGFR, mL/min/1.73 m^2^	76.60 ± 20.85	74.99 ± 20.60^a^	57.91 ± 27.88^a^	<0.001
D-D, mg/L	0.62 (0.42, 1.00)	0.86 (0.55, 2.07)	3.39 (1.11, 8.54)^a,b^	<0.001
FIB, g/L	3.89 ± 1.39	4.08 ± 1.62	3.75 ± 1.57	0.596
PTA, %	97.54 ± 18.56	88.89 ± 21.56^a^	64.96 ± 24.45^a,b^	<0.001
WBC, 10^9^/L	8.80 ± 3.44	10.50 ± 4.56^a^	11.80 ± 5.09^a,b^	<0.001
Neu, 10^9^/L	6.68 ± 3.26	8.7 ± 4.44^a^	9.90 ± 4.73^a,b^	0.000
HGB, g/L	135.41 ± 21.56	136.34 ± 21.91	125.09 ± 26.28^a^	<0.001
ALB, g/L	38.52 ± 5.53	37.58 ± 5.33	33.72 ± 5.43^a^	<0.001
ALT, U/L	27.00 (19.00, 39.00)	93.00 (82.00, 117.00)	345.00 (248.00, 747.25)	<0.001
AST, U/L	31.00 (21.00, 60.00)	87.00 (51.00, 228.00)^a^	410.50 (164.75, 777.75)^a,b^	<0.001
AB, mmol/L	22.90 ± 2.86	21.61 ± 3.92	19.00 ± 6.37	<0.001
mPCO_2_, mmHg	36.97 ± 5.18	36.23 ± 6.63^a^	32.68 ± 6.37^a^	<0.001
mPH	7.40 ± 0.05	7.40 ± 0.07	7.35 ± 0.19^a,b^	<0.001
NPAR	1.97 ± 0.45	2.11 ± 0.51^a^	2.51 ± 0.56^a,b^	<0.001
LVEF	67.00 ± 7.45	65.25 ± 8.14	51.75 ± 6.55	<0.001

Abbreviation: NPAR: Neutrophil percentage-to-albumin ratio, CKMB: Creatine Kinase-MB, isoenzyme, CK: creatine kinase, cTnT: Cardiac Troponin T, NT-proBNP: N-terminal pro-B-type Natriuretic Peptide, hs-CRP: High-sensitivity C-reactive Protein, CHOI: total cholesterol, LDL-C: Low-Density Lipoprotein Cholesterol, HDL-C: High-Density Lipoprotein Cholesterol, Apo A: Apolipoprotein A, Apo B: Apolipoprotein B, HbA1c: Glycated Hemoglobin, Glu: Glucose, Na^+^: Sodium Ion, K^+^: potassium ion, Mg^2+^: Magnesium Ion, P^−^: inorganic phosphate, BUN: blood urea nitrogen, CRE: creatinine, eGFR: estimated glomerular filtration rate, D-D: D-Dimer, FIB: fibrinogen, PTA: prothrombin activity, WBC: white blood cell count, Neu: Neutrophil Count, HGB: hemoglobin, ALB: albumin, ALT: alanine aminotransferase, AST: aspartate aminotransferase, AB: actual bicarbonate, mPCO_2_: measured partial pressure of carbon dioxide, mPH: Measured pH.

### 2.2 NPAR affects the development of acute liver injury

**FIGURE 1:**
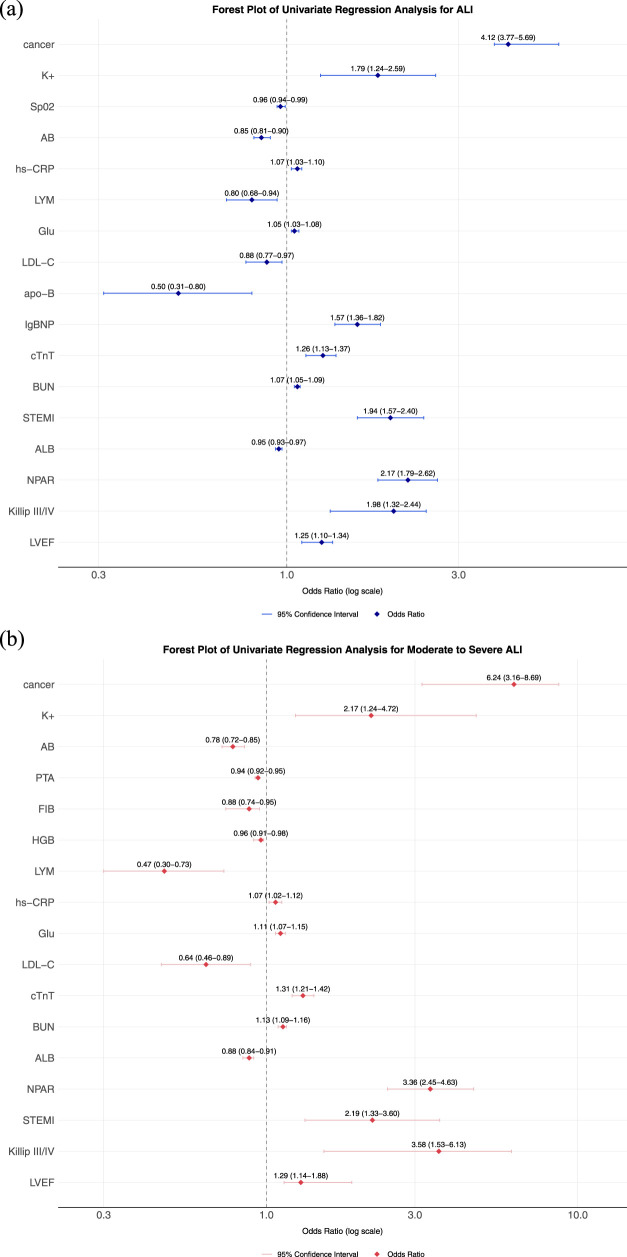
**(a)** Forest plot for univariate Logistic regression analysis of ALI. **(b)** Forest plot for univariate Logistic regression analysis of moderate-to-severe ALI, each horizontal line represents the odds ratio (OR) and 95% confidence interval (CI) for an individual study. The vertical dashed line marks the null effect (OR = 1). Abbreviation: NPAR: Neutrophil percentage-to-albumin ratio, eGFR: estimated glomerular filtration rate, HbA1c: Hemoglobin A1c (Glycated Hemoglobin), CK-MB: Creatine Kinase-MB Isoenzyme, CK: Creatine Kinase, cTnT: Cardiac Troponin T, NT-proBNP: N-terminal pro-B-type Natriuretic Peptide, AB: Actual Bicarbonate, D–D: D-dimer, GLO: Globulin, ALB: Albumin.

Figure 1a presents the binary logistic regression analysis of ALI in AMI patients with T2DM. The results demonstrated that NPAR, cancer, K^+^, SpO_2_, AB, hs−CRP, LYM, Glu, LDL−C, cTnT, BUN, STEMI, Killip classification, and LVEF were identified as independent risk factors for acute liver injury in this population. Table 4 shows the multivariate logistic regression analysis adjusted for relevant factors. After adjustment, NPAR remained an independent risk factor for ALI in T2DM-AMI patients (OR 1.70, 95% CI 1.33–2.17). Figure 1b illustrates the binary logistic regression analysis of acute moderate-to-severe hepatic injury in T2DM-AMI patients. NPAR, cancer, K+, AB, PTA, FIB, HGB, LYM, hs−CRP, Glu, LDL−C, cTnT, BUN, ALB, STEMI, Killip classification, and LVEF were significant predictors for moderate-to-severe hepatic injury. As shown in Table 5, NPAR retained its independent association with acute moderate-to-severe hepatic injury in T2DM-AMI patients in the multivariate analysis (OR 2.90, 95% CI 1.90–4.42).

**TABLE 4:** Multivariable logistic regression analysis of predictors for ALI in T2DM-AMI patients.

Predictors for ALI	Univariate analysis		Multivariate analysis	
	Or (95%CI)	P value	Or (95%CI)	P value
NPAR	2.17 (1.79, 2.62)	<0.001	1.70 (1.33, 2.17)	<0.001
Glu	1.05 (1.03, 1.08)	0.009	1.04 (1.02, 1.06)	0.027
apoB	0.50 (0.31, 0.80)	0.004	0.44 (0.26, 0.73)	0.002
LVEF	1.25 (1.10, 1.34)	<0.001	1.44 (1.21, 1.68)	0.012
hs-CRP	1.07 (1.03, 1.10)	<0.001	-	
Lg (NT-proBNP)	1.57 (1.36, 1.82)	<0.001	-	
K^+^	1.79 (1.24, 2.59)	0.002	-	
AB	0.85 (0.81, 0.90)	0.000	0.94 (0.89, 0.99)	0.032
Cancer	4.12 (3.77, 5.69)	0.000	5.35 (3.95, 8.96)	0.000
cTnT	1.26 (1.13, 1.37)	0.000	1.81 (1.22, 2.68)	0.003
Killip III/IV	1.98 (1.32, 2.44)	<0.001	3.17 (2.32, 4.33)	<0.001
STEMI	1.94 (1.57, 2.40)	<0.001	1.68 (1.30 2.16)	<0.001

Odds ratios (ORs) with 95% confidence intervals (CIs) are reported. Variables with *P* < 0.05 in univariate analysis were included in the initial multivariate model. Final model adjusted for glucose, apolipoprotein B, left ventricular ejection fraction, albumin, cancer history, cardiac troponin T, ST-elevation myocardial infarction, and Killip class III/IV., Dashes (-) indicate variables excluded from the final multivariate model due to collinearity or nonsignificance (*P* ≥ 0.05). All continuous variables were standardized (z-score) prior to analysis.

Abbreviation: NPAR: neutrophil percentage to albumin ratio, Glu: Glucose, apoB: Apolipoprotein B, LVEF: left ventricular ejection fraction, hs-CRP: High-Sensitivity C-Reactive Protein, Lg (NT-proBNP): Logarithm of N-terminal pro-B-type Natriuretic Peptide, K^+^: potassium, AB: albumin, Cancer: Cancer, cTnT: Cardiac Troponin T, Killip III/IV: Killip Class III, or IV, STEMI: ST-Elevation Myocardial Infarction Model: adjusted for Glu, apoB, LVEF, AB, history of Cancer, cTnT, STEMI, and Killip class III/IV.

**TABLE 5:** Multivariable logistic regression analysis of predictors for moderate-to-severe ALI in T2DM-AMI patients.

Predictors for moderate-to-severe ALI	Univariate analysis		Multivariate analysis	
	Or (95%CI)	P value	Or (95%CI)	P value
NPAR	3.36 (2.45, 4.63)	<0.001	2.90 (1.90, 4.42)	<0.001
Cancer	6.24 (3.16, 8.69)	<0.001	5.87 (4.22, 9.12)	<0.001
FIB	0.88 (0.74, 0.95)	0.000	0.76 (0.63, 0.92)	0.005
BUN	1.13 (1.09, 1.16)	0.000	1.10 (1.07, 1.14)	<0.001
AB	0.78 (0.72, 0.85)	0.000	0.84 (0.75, 0.94)	0.001
Killip III/IV	3.58 (1.53, 6.13)	<0.001	3.95 (2.16, 7.22)	0.016
Glu	1.11 (1.07, 1.15)	<0.001	1.06 (1.01, 1.11)	0.010
HGB	0.96 (0.91, 0.98)	<0.001	-	
PTA	0.94 (0.92, 0.96)	0.000	-	

Adjusted odds ratios (ORs) with 95% CIs, are shown. The final model was constructed through backward stepwise selection (retention criterion: *P* < 0.05) and adjusted for cancer history, fibrinogen, blood urea nitrogen, albumin, Killip class III/IV, and glucose. Variables not retained in the final multivariate model are denoted by dashes (-). Continuous predictors were scaled to standard deviation units.

Abbreviation: HbA1c: Hemoglobin A1c, CK-MB: Creatine Kinase-MB, isoenzyme, NPAR: Neutrophil percentage-to-albumin ratio, BUN: blood urea nitrogen, Glu: Glucose, apolipoprotein A, AB: actual bicarbonate, PTA: Prothrombin activity, HGB: hemoglobin.

### 2.3 Cancer is a risk factor for ALI in AMI patients with T2DM

In univariate analysis, cancer was significantly associated with ALI occurrence (OR = 4.12, 95% CI: 3.77–5.69). This association persisted in multivariate analysis after adjusting for Glu, apoB, STEMI, LVEF, AB, cTnT and Killip class (adjusted OR = 5.35, 95% CI: 3.95–8.96). Similarly, cancer independently predicted moderate-to-severe ALI (univariate OR = 6.24, 95% CI: 3.16–8.69; adjusted OR = 5.87, 95% CI: 4.22–9.12). To assess the robustness of this association, we stratified patients by cancer history (with vs. without) and conducted comparative analyses (Table 6).

**TABLE 6:** Comparison of clinical characteristics between patients with and without a history of cancer.

Variables	No history of cancer (n = 5,032)	With history of cancer (n = 101)	P value	AUC (95%CI)	P value	Diagnostic performance
age,year	62.50 ± 11.31	68.01 ± 10.44	<0.001			
male, n (%)	3,837 (76.6%)	78 (77.2%)	0.880			
Arrhythmia, n (%)	828 (16.5%)	21 (20.8%)	0.253			
STEMI, n (%)	2,335 (46.6%)	39 (38.6%)	0.112			
Killip III/IV	526 (10.5%)	8 (7.9%)	0.402			
Hospital stay, day	5.64 ± 2.13	5.45 ± 2.58	0.823			
Diseased vessels			0.054			
Single, n (%)	483 (9.6%)	16 (15.8%)				
Dual, n (%)	1,102 (21.9%)	9 (8.9%)				
Triple, n (%)	3,432 (68.2%)	75 (74.3%)				
FIB	3.88 ± 1.42	4.27 ± 1.53	0.006			
hs-CRP	4.97 ± 3.84	5.66 ± 3.67	0.087			
CKMB, U/L	19.95 (12.41, 49.00)	18.30 (12.51, 38.13)	0.064			
CK, U/L	151.00 (75.00, 511.50)	117.00 (64.00, 347.00)	0.132			
HbA1c, %	8.03 ± 1.75	7.59 ± 1.67	0.017			
LDH, U/L	265.00 (212.00, 377.50)	266.00 (205.50, 365.00)	0.233	0.50 (0.45–0.55)	0.971	No diagnostic value
NPAR	1.98 ± 0.46	2.13 ± 0.65	0.001	ALI: 0.83 (0.79–0.87)	<0.001	Good diagnostic value
				Moderate-to-severe ALI: 0.89 (0.78–0.99)	<0.001	High diagnostic value

Comparison of clinical characteristics and diagnostic performance of NPAR, in T2DM-AMI, patients stratified by cancer history. Values expressed as mean ± standard deviation, median (interquartile range), or n (%) as appropriate. Statistical comparisons: Student’s t-test for normally distributed continuous variables, Mann-Whitney U test for non-normally distributed variables, and χ^2^ test for categorical variables. AUC, area under the curve; ALI, acute liver injury. *P* < 0.05 considered statistically significant.

Abbreviations: ALI: acute liver injury; AUC: area under the curve; CK: creatine kinase; CK-MB: Creatine kinase-MB, isoenzyme; FIB: fibrinogen; HbA1c: Hemoglobin A1c; hs-CRP: High-sensitivity C-reactive protein; LDH: lactate dehydrogenase; NPAR: Neutrophil percentage-to-albumin ratio; STEMI: ST-segment elevation myocardial infarction.

Table 6 revealed that a history of cancer was independently associated with ALI in AMI patients with T2DM. Patients with a cancer history were significantly older (68.01 ± 10.44 vs. 62.50 ± 11.31, *P* < 0.001) and exhibited lower median levels of CK-MB (18.30 [IQR: 12.51–38.13] vs. 19.95 [IQR: 12.41–49.00], *P* = 0.064) and CK (117.00 [IQR: 64.00–347.00] vs. 151.00 [IQR: 79.00–511.50], *P* = 0.132), suggesting reduced myocardial injury severity. Notably, lower rates of Killip class III/IV were observed in the cancer group (8 cases [7.9%]) compared to the non-cancer group (526 cases [10.5%], *P* = 0.402), and a trend toward lower STEMI incidence was noted (39 cases [38.6%] vs. 2,335 cases [46.4%], *P* = 0.112). These findings highlight the critical role of cancer status as an independent predictor of ALI in this high-risk population, with NPAR demonstrating high diagnostic value for moderate-to-severe ALI (AUC = 0.88, 95% CI:0.78–0.99, *P* < 0.001), underscoring the need for tailored clinical management strategies.

### 2.4 ROC analysis of NPAR

**FIGURE 2:**
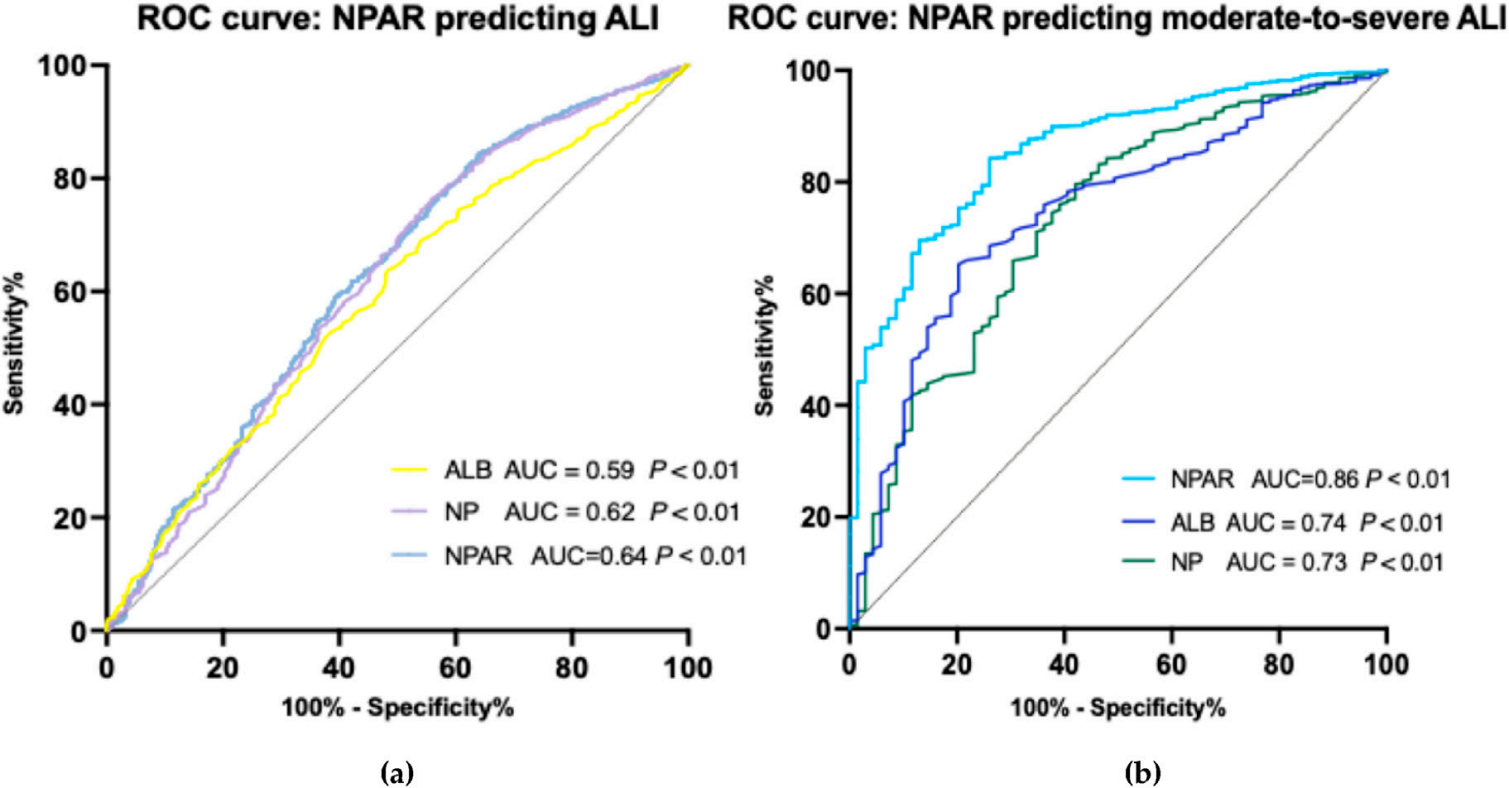
**(a)** Diagnostic performance of NPAR, ALB, and NP for overall acute liver injury prediction in T2DM-AMI cohort. NPAR (red solid line; AUC = 0.64, *P* < 0.01) outperforms ALB (blue dashed line; AUC = 0.59) and NP (green dotted line; AUC = 0.62), though all biomarkers show statistically significant discrimination (*P* < 0.01, DeLong test). Optimal cutoffs annotated at maximal Youden index, cutoff for NPAR predicting ALI is 2.36. Reference line (AUC = 0.5) illustrates random chance. **(b)** Receiver operating characteristic (ROC) curves comparing NPAR, albumin (ALB), and neutrophil percentage (NP) for predicting moderate-to-severe acute liver injury in T2DM-AMI patients. NPAR (red solid line; AUC = 0.86, *P* < 0.01) demonstrates superior discriminative ability versus ALB (blue dashed line; AUC = 0.74) and NP (green dotted line; AUC = 0.73). Diagonal line indicates reference (AUC = 0.5). Optimal cutoffs annotated at maximal Youden index, cutoff for NPAR predicting ALI is 2.91. Statistical significance determined by DeLong’s test (*P* < 0.01 for all comparisons).

ROC analyses of NPAR for ALI and moderate-to-severe ALI in T2DM-AMI patients are presented in Figure 2. For ALI (Figure 2a), NPAR demonstrated predictive performance with an area under the curve (AUC) of 0.63 (*P* < 0.01), which was superior to albumin (ALB, AUC = 0.59, *P* < 0.01) and neutrophil percentage (NP, AUC = 0.62, *P* < 0.01). The optimal NPAR cutoff value for predicting ALI was 2.36 (sensitivity: 78.2%, specificity: 67.0%). Further ROC analysis for moderate-to-severe hepatic injury (Figure 2b) showed that NPAR exhibited a higher AUC of 0.86 (*P* < 0.01), outperforming both ALB (AUC = 0.74, *P* < 0.01) and NP (AUC = 0.73, *P* < 0.01). The optimal NPAR cutoff value for predicting moderate-to-severe hepatic injury was 2.91 (sensitivity: 78.9%, specificity: 93.8%). Additionally, in the cancer patient subgroup, the optimal NPAR cutoff value for predicting ALI was 3.11 (sensitivity: 79.1%, specificity: 92.9%). These findings indicate that NPAR possesses robust discriminative ability for predicting both ALI and moderate-to-severe hepatic injury in this population, and surpasses the individual predictive value of its constituent biomarkers (neutrophil percentage or serum albumin alone). Although patients with chronic liver disease were excluded, the impact of potential confounders like alcohol use or NAFLD was not directly quantified. However, sensitivity analyses using binary covariates (presence/absence) for suspected alcoholic or metabolic liver disease showed minimal fluctuation in the NPAR odds ratio (<8%), supporting the robustness of our findings.

### 2.5 ROC analysis of NPAR in patients with cancer

The ROC analysis demonstrated that NPAR exhibited strong diagnostic performance in predicting ALI among AMI-T2DM patients with cancers. For moderate-to-severe ALI, the AUC of NPAR was 0.89 (*P* = 0.001), with an optimal cut-off value of 3.42, indicating high diagnostic value. For ALI overall, the AUC of NPAR was 0.83 (*P* = 0.001), with the cut-off value of 3.11, further confirming the robust diagnostic accuracy. In contrast, lactate dehydrogenase (LDH) showed no significant diagnostic utility, with an AUC of 0.50 (*P* = 0.971), equivalent to random chance. These results highlight the superior predictive capacity of NPAR over conventional biomarkers like LDH in cancer patients, particularly for severe ALI cases (Table 6).

To summarize, NPAR demonstrates variable diagnostic performance across ALI presentations in T2DM-AMI patients. For overall ALI prediction (cutoff = 2.36), NPAR achieves moderate sensitivity (78.2%; 95% CI: 72.1–83.5) and specificity (67.0%; 61.8–71.9) with limited positive predictive value (PPV = 35.4%; 30.2–41.0) but robust negative predictive value (NPV = 93.0%; 89.6–95.5), yielding a modest AUC of 0.64 (0.59–0.66). Its discriminative ability substantially improves for moderate-to-severe ALI (cutoff = 2.91), where sensitivity remains at 78.9% (70.4–85.8) while specificity increases to 93.8% (90.7–96.1), accompanied by an NPV of 98.6% (96.8–99.5) and AUC of 0.86 (0.82–0.90)—though PPV remains moderate (43.9%; 36.8–51.3). Notably, in cancer patients (cutoff = 3.11), NPAR maintains high sensitivity (79.1%; 71.2–86.2) and specificity (92.9%; 69.9–95.4) with exceptional NPV (97.4%; 93.5–99.6) and an AUC of 0.83 (0.79–0.87), despite suboptimal PPV (39.8%; 31.2–50.8). When predicting moderate-to-severe ALI in T2DM-AMI patients with cancer (cutoff = 3.42), NPAR consistently remain high sensitivity (81.2%; 73.1–89.5) and specificity (94.2%; 72.3–98.1) with exceptional NPV (98.8%; 91.0–99.7) and an AUC of 0.89 (0.78–0.99). Collectively, these findings establish NPAR as a clinically valuable rule-out tool, particularly for excluding moderate-to-severe ALI and ALI in cancer cohorts, while its limited PPV necessitates confirmatory testing for positive results (Supplementary Table 1).

## 3 Discussion

In this study, we explored the relationship between acute hepatic injury and clinical features of AMI patients with T2DM. AMI patients with acute hepatic injury had higher percentage of STEMI, complication of arrhythmia and history of hypertension than those without ALI. They are also older in age and the infarction location is more likely to be the extensive anterior. Our results indicated that NPAR was an independent risk factor for ALI in AMI patients with T2DM. Furthermore, our results showed that a history of cancer is an independent risk factor for ALI in AMI patients with T2DM, and NPAR has good predictive ability for ALI in such patients. These will aid in the early identification of acute liver injury, enabling prompt intervention to improve patients’ clinical outcomes. And it also suggests that we should pay more attention to AMI patients with concomitant cancers and their prognosis in clinical practice.

Our findings establish NPAR as a novel and diagnostically superior biomarker for hepatic injury in patients with T2DM and AMI. In this high-risk cohort—characterized by heightened susceptibility to complications due to synergistic effects of hyperglycemia, insulin resistance, and diabetic microangiopathy—NPAR demonstrated outstanding discriminatory capability for both ALI (AUC = 0.64, *P* < 0.01) and moderate-to-severe ALI (AUC = 0.86, *P* < 0.01). This performance significantly surpassed established individual markers (albumin, neutrophil percentage). Critically, NPAR emerged as an independent risk factor for ALI after rigorous adjustment for renal function and coagulation parameters, a robustness attributable to its unique integration of two fundamental pathophysiological axes: systemic inflammation (reflected by neutrophil percentage) and metabolic/nutritional stress (indicated by albumin). This composite design captures the clinical phenotype of T2DM-AMI patients with ALI—typically older, with higher rates of STEMI, anterior infarction, arrhythmias, and hypertension—while mechanistically explaining the detrimental interplay between neutrophilic activation (e.g., myeloperoxidase-driven oxidative stress) and hypoalbuminemia (reflecting hepatic synthetic impairment and diminished antioxidant capacity).

The biological coherence of NPAR is further evidenced by its predictive consistency across diverse pathophysiological contexts. Beyond its core link to inflammation-nutrition dysregulation in T2DM-AMI—where chronic hyperglycemia accelerates hepatic lipid dysregulation and Kupffer cell activation—NPAR maintained diagnostic power in cancer-bearing subpopulations (AUC = 0.83), despite their paradoxical reduction in cardiac enzymes (CK/CK-MB). This phenomenon may arise from cancer-associated coagulopathy impairing thrombus formation or metabolic dysregulation shifting cell death toward apoptosis over necrosis. Such context-dependent adaptability underscores role of NPAR as a generalized indicator of systemic injury. Importantly, its elevation aligns with key drivers identified in ALI cohorts: hyperinflammatory markers (hs-CRP, fibrinogen), metabolic disruptors (glucose, D-dimer), and perturbations in organ crosstalk (inverse correlations of LDL-C, eGFR, and albumin with injury severity). The 7.60% incidence of ALI in AMI—though seemingly modest—carries profound prognostic weight, emphasizing the clinical imperative for early detection in an era where hepatic surveillance remains neglected despite improved AMI workflows.

Crucially, NPAR’s actionable thresholds (≥2.36 for ALI; ≥2.91 for moderate-to-severe injury) enable precision risk stratification directly addressing unmet clinical needs. This threshold effect, potentially J-shaped as in diabetes mortality studies, signifies a tipping point where cumulative inflammation-nutrition burden overwhelms compensation. Its independence from HbA1c/CK-MB in multivariable models positions NPAR as a vital supplement to current risk frameworks—particularly relevant given the therapeutic gaps observed: underutilization of guideline-directed lipid therapy in ALI groups (45.0% vs. 96.2% in non-injured, *P* < 0.001) due to hepatotoxicity concerns, and excessive diuretic use exacerbating renal dysfunction. Integration of NPAR into clinical workflows could mitigate these pitfalls by guiding: (1) intensified hepatic monitoring in high-risk subgroups (e.g., cancer patients with elevated NPAR/FIB), (2) nutritional/anti-inflammatory interventions to break the injury cascade, and (3) medication optimization (e.g., hepatoprotective agents or dose-adjusted anticoagulants). Future studies should explore NPAR’s utility in targeting gut-liver axis dysregulation—a mechanism implicated in fibrosis progression—to transform mechanistic insights into therapeutic innovation.

Moreover, our study identified a history of cancer as an independent risk factor for ALI in AMI patients with T2DM. Notably, NPAR also demonstrated strong predictive capacity for ALI in this subgroup. These findings underscore the potential of NPAR as a robust biomarker for detecting cancer-associated ALI. However, the generalizability of these results is limited by the relatively small sample size and the lack of stratification by cancer type. Expanding the cohort to include diverse malignancies will be critical to validate the efficacy of NPAR across different cancer subtypes and stages, particularly given the heterogeneity in metabolic and inflammatory profiles among cancers (Pearson-Stuttard et al., 2021). NPAR can effectively predict the risk of cardio-hepatic injury in AMI and T2DM patients with comorbid cancers. Cancers increase neutrophil proportion through three mechanisms of “inflammatory cytokine release + metabolic dysfunction + treatment toxicity”: Inflammatory cytokines (IL-6, TNF-α) activate systemic inflammation and exacerbate cardio-hepatic interactive injury; Insulin resistance induced by tumors and diabetes inhibits hepatic albumin synthesis; Anthracyclines, anti-VEGF drugs, etc., superimposed on AMI-induced ischemia and hypoxia, trigger dual injury to cardio-hepatic microcirculation. Diabetes further reduces the efficiency of hepatocyte albumin synthesis, making NPAR a comprehensive indicator reflecting the “myocardial ischemia-hepatic inflammation-metabolic imbalance” pathological network. Clinical dynamic monitoring of NPAR helps early identify high-risk populations. Intervention strategies include optimizing cardiovascular support, adjusting anti-tumor regimens (such as replacing cardiotoxic drugs), and nutritional interventions, so as to block the pathological chain of “tumor inflammation→cardio-hepatic injury→metabolic imbalance” and provide a basis for interdisciplinary treatment.

In AMI patients complicated by T2DM, the presence of cancer significantly increases the risk of ALI. The mechanisms involve both the tumor’s own pathological effects—such as secreting inflammatory cytokines like IL-6 and TNF-α, which synergize with T2DM-induced insulin resistance to exacerbate hepatic oxidative stress, and space-occupying effects that directly impair hepatic blood flow or trigger “aseptic hepatitis”—and the multi-target hepatotoxicity of anticancer drugs. For example,: Anthracyclines (e.g., doxorubicin) damage mitochondria in both cardiac and hepatic cells by inhibiting topoisomerase IIβ and inducing free radical generation, leading to cardiotoxicity (dose-dependent cardiomyopathy) and hepatotoxicity (hepatocyte necrosis) through shared oxidative stress mechanisms (Wu et al., 2022; Balough et al., 2025). Platinum-based drugs (e.g., cisplatin) target proximal tubule epithelial cells in the liver, inducing cholestasis via p53-mediated apoptosis and ATPase inhibition, which forms a “toxic cascade” with AMI-induced hepatic ischemia and hypoxia (Xu et al., 2021). Anti-VEGF/VEGFR agents (e.g., bevacizumab) disrupt the integrity of hepatic sinusoidal endothelial cells, inducing hepatic sinusoidal obstruction syndrome (HSOS), while reducing myocardial microcirculatory perfusion and exacerbating “cardio-hepatic ischemic cross-injury. (Yıldırım et al., 2023).” EGFR inhibitors (e.g., erlotinib) block hepatocyte regeneration signaling pathways (e.g., RAS-RAF-MEK-ERK), further impairing repair capacity in the context of T2DM (Bak et al., 2025). mTOR inhibitors (e.g., everolimus) inhibit hepatic lipid metabolism genes (e.g., PPARα, AMPK), exacerbating hepatic steatosis in T2DM patients. PD-1/PD-L1 inhibitors may trigger autoimmune hepatitis (incidence: 5%–10%) via CD8^+^ T cell-mediated hepatotoxicity, synergizing with the tumor’s systemic inflammation to activate intrahepatic immune injury. Additionally, hypoalbuminemia common in cancer patients weakens hepatic antioxidant defenses, and the “inflammation-nutrition imbalance” marker NPAR (neutrophil percentage/serum albumin) demonstrates high efficiency in predicting ALI (AUC = 0.89–0.93). Future strategies should focus on developing hepatoprotectants targeting specific drug receptors and optimizing personalized prevention by integrating NPAR and target-related biomarkers.

A key limitation of this observational study is the inability to establish causal relationships between NPAR and hepatic injury, necessitating validation through experimental models (e.g., animal studies or *in vitro* systems) to determine whether NPAR directly mediates hepatic damage. Furthermore, our analysis of cancer patients lacks stratification by malignancy type—including distinctions between solid tumors (e.g., lung/breast cancers) and hematologic malignancies (e.g., leukemia/lymphoma)—which may mask subtype-specific risks given their divergent pathogenic mechanisms, tumor burdens, and impacts on systemic inflammation/nutritional status. Critically, the absence of granular data on anticancer regimens (e.g., drug types, doses, duration) precludes assessment of treatment-specific hepatotoxicity from chemotherapy, targeted agents, immunotherapy, or concomitant hepatotoxic medications. This gap necessitates caution in extrapolating utility of NPAR to drug-induced liver injury scenarios. Future studies incorporating cancer-type stratification and detailed treatment metadata will enable more accurate evaluation of NPAR’s association with hepatic injury while controlling for oncotherapy-related confounders, thereby generating more robust clinical insights.

## 4 Conclusion

NPAR demonstrates sound predictive efficacy for moderate-to-severe hepatic injury in AMI patients with T2DM and may serve as a viable early predictive marker for clinical monitoring. Cancer is an independent risk factor for ALI in this patient population, and NPAR retains strong predictive capacity for ALI even in the presence of concomitant cancer.

## 5 Methods

### 5.1 Study population

This study aimed at conducting a retrospective analysis of patients diagnosed with AMI and T2DM and admitted in the First Affiliated Hospital of Xi’an Jiaotong University from January 2018 to May 2025. Data of clinical characteristics, lab tests and coronary angiography conclusion are obtained from the electronic medical record system of hospital biospecimen information resource center. The study protocol received approval from the Medical Ethics Committee of the First Affiliated Hospital of Xi’an Jiaotong University. This study set these inclusion criteria: 1) age ≥18 years; 2) meeting the diagnostic criteria for AMI according to the 2018 ESC/ACC/AHA/WHF Myocardial Infarction 4th Edition Global Definition; 3) patients with type 2 diabetes mellitus (T2DM); and 4) underwent liver function testing during admission and complete baseline data. The exclusion criteria were as follows: 1) Patients with pre-existing chronic liver diseases (e.g., hepatitis, hepatic steatosis, cirrhosis); 2) Patients comorbid with psychiatric disorders, or autoimmune diseases; 3) Patients with hematopoietic proliferative disorders or hyperthyroidism; 4) Patients with hospitalization duration <24 h; 5) Patients with incomplete clinical data.

According to alanine transaminase (ALT) level, patients were divided into mild hepatic injury group (2 ULN ≤ ALT < 5ULN) (Kim et al., 2008), moderate-to-severe hepatic injury group (ALT ≥ 5ULN) and the non-hepatic injury group (ALT < 2ULN) (Ichai et al., 2019; European Association for the Study of the Liver, 2019). For patients suspected of having alcoholic or metabolic liver disease, binary covariates were included for sensitivity analysis.

### 5.2 Definitions and outcome

**FIGURE 3:**
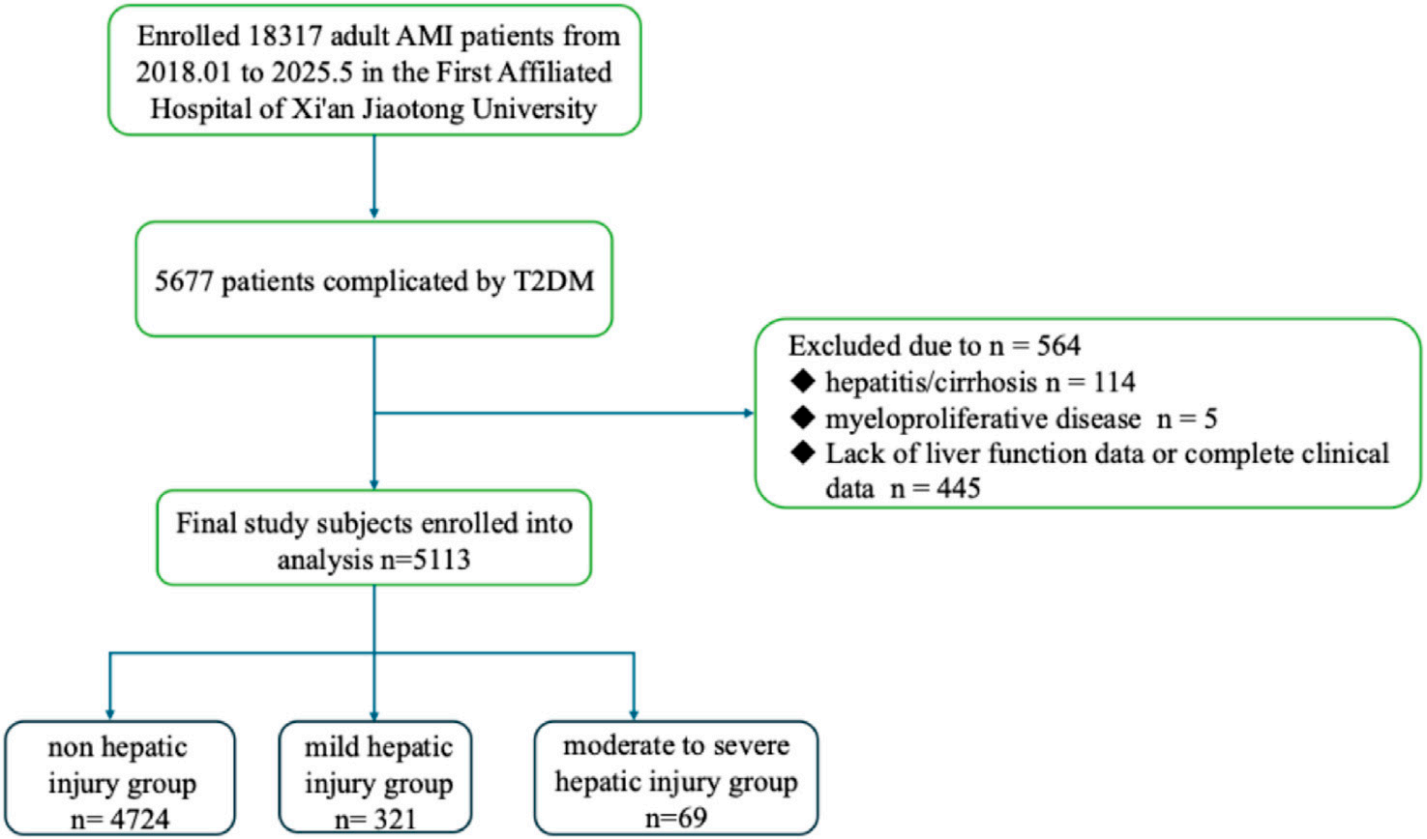
Flowchart of the enrolled patients.

AMI should be considered when myocardial cell apoptosis is congruent with the clinical presentations of myocardial ischemia, and fluctuations in cardiac biomarker levels, particularly high-sensitivity cardiac troponin (hs-cTn) T or I, are ascertainable, with at least one concentration exceeding the 99th percentile of the upper reference limit. ALT can specifically reflect liver dysfunction, as elevated aspartate aminotransferase (AST) has been shown in the case of ischemia-cell death of other tissues, including the kidney, skeletal muscle and brain. Given the greater specificity of ALT than AST, ALT was eventually chosen to evaluate acute liver injury. Acute liver injury was defined as ALT > 2ULN on the first test of liver function on admission, without known cause for previous hepatic injury (European Association for the Study of the Liver, 2019; Little et al., 2019). Non-hepatic injury was defined as ALT < 2ULN, mild hepatic injury was defined as 2ULN ≤ ALT < 5ULN and moderate-to-severe hepatic injury was defined as ALT ≥ 5ULN (Sarin et al., 2019; Ribeiro et al., 2020). NPAR was calculated as the ratio of neutrophil percentage to serum albumin level (NPAR = NP/ALB). The primary outcome is the onset of acute liver injury. Figure 3 shows the flow chart of the study.

### 5.3 Statistical analysis

A Continuous variables were tested for normality using the Kolmogorov-Smirnov test; information that met normal distribution was expressed as mean ± standard deviation, and non-normally distributed variables were expressed as interquartile range, and two-group comparisons were made using the t-test. For ordered categorical variables between two groups, the Mann-Whitney U test was used for comparison; for unordered multicategorical variables between two groups, the Chi-square test was applied. Univariate and multivariate logistic regression analyses were used to test the association between predictive indicators and AKI. All candidate variables with potential relevance to the outcome (e.g., demographic, clinical, and laboratory factors) are included in the univariate analysis. Each variable undergoes separate logistic regression to assess its crude association with the outcome, yielding OR, 95% CI, and p-value. Variables with *P* < 0.1 in univariate analysis or clinical relevance were included in multivariable models. Variables were initially selected based on literature and guidelines. After univariate screening (*P* < 0.10), 8 predictors were finally identified through bidirectional stepwise regression (AIC criterion). The stability of the model was ensured by the variance inflation factor (VIF <2). Six preset interaction terms were tested (*P* > 0.05). Missing data were handled using multiple imputation. Categorical variables were expressed as percentages N (%), and comparisons between groups were made using the χ^2^ test. Data analyses were performed using the SPSS (version 26.0 package (IBM, Armonk, NY, United States). Figures were plotted using GraphPad Prism 9.0 (GraphPad Prism Software Inc., San Diego, CA, United States). *P* < 0.05 was considered statistically significant.

## Data Availability

The original contributions presented in the study are included in the article/Supplementary Material, further inquiries can be directed to the corresponding author.

## Appendix

**TABLE A1:** Diagnostic Performance of NPAR in Predicting ALI in T2DM-AMI Patients.

	NPAR Cutoff	Sensitivity (%)	Specificity (%)	PPV (%)	NPV (%)	AUC (95% CI)
Overall ALI	2.36	78.2 (72.1, 83.5)	67.0 (61.8, 71.9)	35.4 (30.2, 41.0)	93.0 (89.6, 95.5)	0.64 (0.59, 0.66)
Moderate to Severe ALI	2.91	78.9 (70.4, 85.8)	93.8 (90.7, 96.1)	43.9 (36.8, 51.3)	98.6 (96.8, 99.5)	0.86 (0.82, 0.90)
ALI in Patients with Cancer	3.11	79.1 (71.2, 86.2)	92.9 (69.9, 95.4)	39.8 (31.2, 50.8)	97.4 (93.5, 99.6)	0.83 (0.79, 0.87)
Moderate to Severe ALI in Patients with Cancer	3.42	81.2 (73.1, 89.5)	94.2 (72.3, 98.1)	44.1 (36.5, 49.6)	98.8 (91.0, 99.7)	0.89 (0.78, 0.99)

Abbreviation: PPV, positive predictive value; NPV, negative predictive value; AUC, area under the receiver operating characteristic curve; CI, confidence interval. Diagnostic performance metrics are presented alongside their respective 95% confidence intervals.
