# Supplementary material for: Inflammatory-nutritional duality of NPAR: a novel biomarker for early prediction of acute liver injury in acute myocardial infarction complicated by type 2 diabetes mellitus
Source: Front Pharmacol. 2025 Sep 2;16:1643871. doi: 10.3389/fphar.2025.1643871 (PMC12438973; doi:10.3389/fphar.2025.1643871)
Supplement: Supplementary file 1 [file Table1.docx]

Appendix Table 1: Diagnostic Performance of NPAR in Predicting ALI in T2DM-AMI Patients

|  | NPAR  Cutoff | **Sensitivity (%)** | **Specificity (%)** | **PPV (%)** | **NPV (%)** | **AUC (95% CI)** |
| --- | --- | --- | --- | --- | --- | --- |
| Overall ALI | 2.36 | 78.2(72.1, 83.5) | 67.0 (61.8, 71.9) | 35.4(30.2, 41.0) | 93.0 (89.6, 95.5) | 0.64(0.59, 0.66) |
| Moderate to Severe ALI | 2.91 | 78.9(70.4, 85.8) | 93.8 (90.7, 96.1) | 43.9 (36.8, 51.3) | 98.6(96.8, 99.5) | 0.86 (0.82, 0.90) |
| ALI in Patients with Cancer | 3.11 | 79.1(71.2, 86.2) | 92.9 (69.9, 95.4) | 39.8 (31.2, 50.8) | 97.4 (93.5, 99.6) | 0.83 (0.79, 0.87) |
| Moderate to Severe ALI in Patients with Cancer | 3.42 | 81.2(73.1, 89.5) | 94.2(72.3, 98.1) | 44.1(36.5, 49.6) | 98.8(91.0, 99.7) | 0.89(0.78, 0.99) |

 Abbreviation: PPV: positive predictive value; NPV: negative predictive value; AUC: area under the receiver operating characteristic curve; CI: confidence interval. Diagnostic performance metrics are presented alongside their respective 95% confidence intervals.
